# Improvement of *Bemisia tabaci* (Hemiptera: Aleyrodidae) Fitness on Chinese Kale upon Simultaneous Herbivory by *Plutella xylostella* (Lepidoptera: Plutellidae)

**DOI:** 10.3390/biology11010072

**Published:** 2022-01-04

**Authors:** Jun Jiang, Li-Li Xu, Wen-Yuan Yu, Shi-Ze Zhang, Tong-Xian Liu

**Affiliations:** 1 State Key Laboratory of Crop Stress Biology for Arid Areas, Northwest A&F University, Yangling 712100, China; junjiang@nwafu.edu.cn (J.J.); xu-lili@nwafu.edu.cn (L.-L.X.); yuwenyaun@nwafu.edu.cn (W.-Y.Y.); txliu@nwafu.edu.cn (T.-X.L.); 2 Key Laboratory of Entomology and Pest Control Engineering, College of Plant Protection, Southwest University, Chongqing 400716, China

**Keywords:** cotton whitefly, diamondback moth, fitness, heterospecific interaction, plant-mediated insect interaction, performance, population dynamics

## Abstract

**Simple Summary:**

Different herbivores feeding on the same plant can interact through plant-mediated effects. Cotton whitefly and diamondback moth are two of the most destructive pests in the world, and they often occur together in cruciferous plants. However, how the performance and fitness of them are affected when co-occurring in the same host plant remains unclear. The present study demonstrates that cotton whitefly has become a dominant competitor by gaining increased fitness benefits when it is mixed with DBM on the same host plant irrespective of sequences of their arrival, which may be one of the reasons for the rapid expansion and outbreak of the whitefly population worldwide.

**Abstract:**

*Bemisia tabaci* and the diamondback moth (DBM), *Plutella xylostella*, are two major cosmopolitan pests that often occur together and cause severe economic losses to cruciferous crops. However, little is known about how they interact with each other. To determine the effects of defense responses induced by the two pests on the biology and population dynamics of the herbivores, we studied the performance and fitness of *B. tabaci* and DBM when they damaged Chinese kale simultaneously and in different orders. The results showed that DBM pre-infestation shortened the developmental duration, increased longevity, oviposition days, and fecundity of *B. tabaci*. Meanwhile, the intrinsic rate of increase (*r*), net reproductive rate (*R*_0_) and finite rate of increase (*λ*) of *B. tabaci* increased significantly with dual infection as compared with only *B. tabaci* infestation. In contrast, *B. tabaci* pre-infestation reduced the longevity and oviposition days of DBM, but the population parameters *r*, *R*_0_, and *λ* did not vary significantly compared with only DBM infestation. Thus, co-infestation of *B. tabaci* and DBM was beneficial to the performance of the *B. tabaci* population. The present findings highlight that *B. tabaci* has become a dominant competitor when mixing with DBM on the same host plant.

## 1. Introduction

In ecological systems, plants are often infested by various herbivorous insects at the same time or at different times [1,2,3]. Damage by herbivores can induce changes in the morphological characteristics and physiological metabolism of host plants [4,5]. The changes in host plants can affect host selection, survival, fecundity, and population dynamics of herbivores, as well as their interactions [6,7,8]. Phytochemical defense responses against herbivores are often herbivore-specific and depend to some extent on the feeding mode of herbivores. In general, leaf-chewing insects induce jasmonic acid (JA) regulated defense, whereas phloem feeders induce salicylic acid (SA) regulated defense [9,10]. The induction of JA- and SA-related defense compounds in plants and the plant-mediated indirect interactions between herbivores differ, which have an impact on herbivore performance. The growth and development of herbivores are often negatively affected when the involved insects belong to the same feeding guilds but positive for subsequent herbivore performance when they belong to different feeding guilds [11,12,13,14]. However, there have been inconsistent reports. The aphid *Aphis nerii* Boyer de Fonscolombe (Hemiptera: Aphididae) develops more slowly on milkweed plants *Asclepias syriaca* L. and *A. tuberosa* L. (Apocynaceae) pre-infested with the monarch caterpillars *Danaus plexippus* L. (Lepidoptera: Nymphalidae) [15]. In a recent study, it has been reported that early arriving herbivores negatively affect subsequent insect developmental duration and population growth when the involved insects are from different feeding guilds [16]. Therefore, plant quality-mediated interactions between herbivores may not only depend on the combination of attackers but also on their sequence of arrival. Moreover, many herbivores have multiple generations throughout the season and are not restricted to a single sequence of arrival on a shared host plant. Therefore, it is possible that different herbivorous insect species simultaneously damage the same plant. This may lead to complex interactions between different insect species that are derived from physiological and metabolic changes in the plants [17,18,19]. Exploring interspecific interactions across different herbivores can allow us to understand how herbivore performance is affected when herbivores from different feeding guilds co-occur on the same host plant.

The cotton whitefly *Bemisia tabaci* (Gennadius) (Hemiptera: Aleyrodidae), a pest with piercing-sucking mouthparts, and the diamondback moth (DBM), *Plutella xylostella* (L.) (Lepidoptera: Plutellidae), a pest with leaf-chewing mouthparts, are two major cosmopolitan pests that attack cruciferous crops [20,21]. During a preliminary investigation, we found that *B. tabaci* and DBM usually feed on cruciferous vegetables together in a mixed-population pattern. It is well known that *B. tabaci* induces SA-dependent defense responses [22], and DBM larvae induce JA-dependent defense responses [23]. Some studies have shown that the glucosinolate content and nutritional quality of cruciferous plants are upregulated and downregulated, respectively, when they are infested by DBM and *B. tabaci* [24,25,26]. Since the plant defense responses induced by *B. tabaci* and DBM are different, their mixed occurrence may lead to the interaction of plant defense responses, resulting in different effects on the performance and fitness of the two herbivores. To date, however, how the performance and fitness of *B. tabaci* and DBM are affected when they co-occur in the same host plant remains unclear.

It is known that *B. tabaci* and DBM can induce different defense signaling pathways, and then differential changes in plant chemistry can occur. We hypothesized that these changes in host plants might result in a facilitative or competitive advantages for *B. tabaci* or/and DBM, which may affect their performance and population dynamics. Therefore, in this study, we addressed this knowledge gap by using the age-stage, two-sex life table on the interaction between two different feeding guilds, *B. tabaci* and DBM, mediated by their host plant Chinese kale. The primary objective of this study was to investigate whether *B. tabaci* and/or DBM benefit when feeding on the same host plant at the same time or at different times. To this end, we first compared the survival, growth, development, fecundity and longevity of *B. tabaci* and DBM feeding on plants pre-infested by heterospecific individuals or co-infested by conspecific and heterospecific individuals. Second, we compared the population parameters, including the finite rate of increase (*λ*), intrinsic rate of increase (*r*), net reproductive rate (*R*_0_), and mean generation time (*T*) using the age-stage, two-sex life table. The current study provides a comprehensive insight into the interaction between herbivores from two different feeding guilds co-feeding on one host plant, and the data can also be used to predict the population growth and dynamics of *B. tabaci* and DBM in the field. Our findings suggest that *B. tabaci* population has become a dominant competitor when it is mixed with DBM on the same host plant, which may be one of the reasons for the rapid expansion and outbreak of *B. tabaci* population worldwide.

## 2. Materials and Methods

### 2.1. Host Plant and Insect Cultures

Seeds of Chinese kale (*Brassica alboglabra* var. *alboglabra* Bailey cv. Zhonghuajianye) were purchased from Yangling Nongcheng Seed Supplement Company (China) and sown in a rectangular pot (465 × 175 × 165 mm) containing a mixture of commercial peat moss (Pindstrup Mosebrug A/S, Ryomgaard, Denmark), perlite, and buldymite at 3:1:1 by volume in climate-controlled growth chambers (RXZ-600C, Ningbo Jiangnan, China) at 25 ± 1 °C, 60–80% relative humidity (RH), and a photoperiod of 16L:8D. After full expansion of the 2–3 true leaves, the seedlings were selected for uniformity and transplanted into individual plastic pots (diameter, 120 mm) containing the above-mentioned mixture and watered every 3 days. The plants were used in all experiments after full expansion of the fifth and sixth leaves (approximately 45–50 days old).

*Bemisia tabaci* and DBM were collected from cabbage mustard (*Brassica alboglabra* Bailey) plants in a greenhouse in Yangling, Shaanxi, China. *Bemisia tabaci* was identified as *B. tabaci* Middle East-Asia Minor 1 (MEAM1) using a random amplified polymorphic DNA-polymerase chain reaction with the mitochondrial C oxidase subunit Ⅰ gene [27]. *Bemisia tabaci* and DBM were maintained separately on Chinese kale in nylon mesh covered cages (60 × 60 × 60 cm) in an insectary maintained at 25 ± 2 °C, 60–80% relative humidity (RH), and a photoperiod of 16L:8D. Three cages were used to establish separately *B. tabaci* or DBM cultures. Each cage had four potted Chinese kale plants of 5–6-weeks-old, and about 100 pairs *B. tabaci* adults or 30 pairs DBM adults (<24 h old) were released into cages. A cotton ball soaked with 10% sucrose water was used to supply nutrition for DBM adults. The plants were watered every three days and replaced with fresh plants as needed. It took about 19–25 days for *B. tabaci* to complete one generation, and 24–30 days for DBM to complete one generation. About 100 pairs *B. tabaci* or 30 pairs DBM adults collected in the field were mixed into their respective breeding populations every three generations to avoid the deleterious effects of inbreeding. *Bemisia tabaci* and DBM cultures were reared for more than five generations before use in the experiments.

### 2.2. Bemisia Tabaci Performance

**Figure 1:**
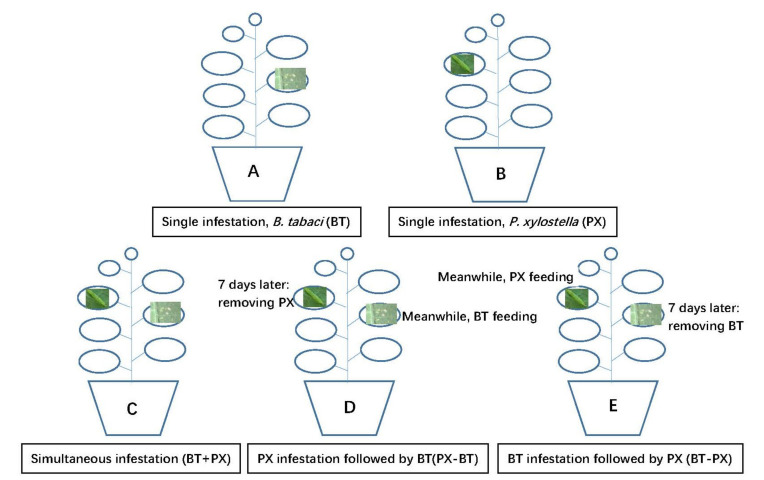
Schematic illustration of experimental setup. *Bemisia tabaci* (BT) and *Plutella xylostella* (PX) performance and fitness benefits were evaluated when feeding as single herbivore on Chinese kale (**A**,**B**, respectively), on plants with prior herbivore damage (**D**,**E**, respectively) and with simultaneous damage (**C**).

To evaluate herbivore performance, *B. tabaci* adults were added to plants that were assigned one of the following three treatments (Figure 1A,C,D): (1) Single herbivory (i.e., *B. tabaci* infestation alone, BT)—20 pairs of newly emerged whitefly adults (<24 h old) from the insectary were collected and released into a leaf-clip cage (diameter, 4 cm; height, 2 cm) on the undersurface of the fourth leaf of Chinese kale. The edge of the leaf-clip cage was covered with a sponge to prevent mechanical wounds on the leaf. The whitefly adults were allowed to lay eggs for 2 h before being removed. Approximately 7 days later, most of the *B. tabaci* eggs hatched into the first instar nymphs. (2) Simultaneous herbivory (i.e., *B. tabaci* and DBM simultaneous infestation, BT + PX)—20 pairs of newly emerged whitefly adults (<24 h old) from the insectary were collected and released into the same leaf-clip cage on the undersurface of the fourth leaf of Chinese kale. The whitefly adults were allowed to lay eggs for 2 h before being removed. After 7 days, when most of the *B. tabaci* eggs hatched into the first instar nymphs, two newly hatched first instar larvae of DBM (<6 h old) were introduced onto the fifth leaf on the same plant and this leaf was encased in a small nylon-mesh bag (length,10 cm; width, 8 cm). The opening of the bag was closed with a fine zipper. (3) Prior herbivory (i.e., DBM infestation followed by *B. tabaci*, PX − BT)—20 pairs of newly emerged whitefly adults (<24 h old) from the insectary were collected and released into the same leaf-clip cage on the undersurface of the fourth leaf. After 2 h, the clip-cage and whitefly adults were removed from the plants. Meanwhile, two newly hatched first instar larvae of DBM (<6 h old) were introduced to the fifth leaf on the same plant and this leaf was encased in the same small nylon-mesh bag. Approximately 7 days later, the DBM larvae were removed. At this time, most of the whitefly eggs hatched into first instar nymphs. Six plants (one plant represented a replication) from each treatment were used in the experiments.

When most of the *B. tabaci* eggs hatched into the first instar nymphs, that is, 7 days later, about 30 first instar nymphs were left, and others were removed with the help of a stereomicroscope. The duration of development and survival for each immature stage of *B. tabaci* were examined daily using a stereomicroscope until they reached the adult stage. In order to study the reproduction, a pair of newly emerged adult whiteflies (<24 h old) was placed on the lower surface of the leaf enclosed in a clip cage in a different plant. The number of eggs laid by females daily was counted using a dissecting microscope after the pair and clip cage were transferred carefully to another new leaf from a different plant. A total of ten pairs of newly emerged adult whiteflies (<24 h old) from each treatment were used in the experiments, and each treatment was repeated three times. These observations continued until the death of female insects. All experiments were carried out in climate growth chambers (RXZ-600C, Ningbo Jiangnan, China) at 25 ± 1 °C and 60–80% RH and a photoperiod of 16L:8D.

### 2.3. Plutella Xylostella Performance

A similar bioassay was designed to evaluate the developmental time and survival of DBM in the presence and absence of whitefly infestation (Figure 1B,C,E): (1) Single herbivory (i.e., DBM larvae feeding alone, PX)—Two newly hatched first instar larvae (<6 h old) were introduced onto the fifth leaf encased in a nylon-mesh bag. (2) Simultaneous herbivory (PX + BT)—This treatment was the same as that of BT + PX in 2.2. (3) Prior herbivory (BT − PX)—This treatment was the same as that of BT in 2.2. When most of the *B. tabaci* eggs hatched into the first instar nymphs, about 30 first instar nymphs were left, and others were removed with the help of a stereomicroscope. After 7 days, the *B. tabaci* nymphs were removed. Meanwhile, two newly hatched first instar larvae of DBM (<6 h old) were introduced onto the fifth leaf and this leaf was encased in a small nylon-mesh bag. Ten plants (one plant represented a replication) from each treatment were used in the experiments.

The developmental time and survival rate of DBM were examined daily. In order to study reproduction, the males and females were paired after the adults emerged (<24 h old). The pair was released onto an undamaged new leaf encased in a small nylon-mesh bag (length,10 cm; width, 8 cm) in a different plant, and a small cotton ball dipped in 10% sucrose solution was placed into the bag to provide nutrition. The number of eggs laid in the leaf was counted daily after the adults were transferred to a new leaf every day. Meanwhile, the small cotton ball was also replaced by a new one. A total of six pairs of newly emerged DBM adults from each treatment were used in the experiments, and each treatment was repeated three times. These observations continued until the death of the females. All experiments were carried out in climate-controlled growth chambers (RXZ-600C, Ningbo Jiangnan, China) at 25 ± 1 °C, 60–80% RH, and a photoperiod of 16L:8D.

### 2.4. Statistical Analysis

The raw life table data of individual *B. tabaci* and DBM were analyzed using the TWO-SEX-MSChart program [28], based on the age-stage, two-sex life table theory [29] and the method described by Chi [30]. The survival rate (*s_xj_*) (*x* = age, *j* = stage), which is the probability that a newly laid egg survives to age *x* and stage *j*, and fecundity *f_xj_*, which is the number of hatched eggs produced by an adult female at age *x*, were calculated. Age-specific survival rate (*l_x_*) was calculated as $l_{x}=\overset{m}{\underset{j=1}{\sum }}s_{xj}$, where m is the number of stages. Age-specific fecundity (*m_x_*) was calculated as $m_{x}=\overset{m}{\underset{j=1}{\sum }}s_{xj}f_{xj}/\overset{m}{\underset{j=1}{\sum }}s_{xj}$. The net reproductive rate, which is defined as the total number of offspring that an individual can produce during its lifetime, is calculated as $R_{0}=\overset{\infty }{\underset{x=0}{\sum }}l_{x}m_{x}$. The intrinsic rate of increase was calculated using the Lotka–Euler equation with age indexed from 0 as $\overset{\infty }{\underset{x=0}{\sum }}e^{-r\left(x+1\right)}l_{x}m_{x}=1$. The mean generation time represents the period that a population requires to increase to *R*_0_-fold of its size as time approaches infinity and the population settles down to a stable age-stage distribution. Mean generation time is calculated as $T=\frac{\mathrm{In}R_{0}}{r}$. Age-stage-specific life expectancy (*e_xy_*) (i.e., the time that an individual of age *x* and stage *y* is expected to live) was calculated as $e_{xy}=\overset{n}{\underset{i=x}{\sum }}\overset{m}{\underset{j=y}{\sum }}s_{ij}^{\prime }$, where $s_{ij}^{\prime }$ is the probability that an individual of age *x* and stage *y* will survive to age *i* and stage *j*. In the age-stage, two-sex life table, it is calculated as $v_{xy}=\frac{e^{r\left(x+1\right)}}{s_{xy}}\overset{n}{\underset{i=x}{\sum }}e^{-r\left(i+1\right)}\overset{m}{\underset{j=y}{\sum }}s_{ij}^{\prime }f_{ij}$.

The means and standard errors of developmental time, pre-oviposition period, adult longevity, fecundity, oviposition day, total preoviposition period, sex ratio, and population parameters, i.e., the finite rate of increase (*λ*), intrinsic rate of increase (*r*), net reproductive rate (*R*_0_), and mean generation time (*T*) of *B. tabaci* or DBM for each treatment, were calculated using the bootstrap method with 100,000 replicates, and the differences among treatments were measured and compared using the Tukey–Kramer procedure with a significance level of *p* < 0.05.

## 3. Results

### 3.1. Performance of B. tabaci

The egg-adult (from embryonic development to adult emergence) developmental duration of *B. tabaci* was 7.9% and 6.2% lower in plants that were pre-infested with DBM (PX − BT treatment) or simultaneously exposed to whiteflies plus DBM (PX + BT treatment) than in plants infested with whiteflies only (BT, control treatment) (*p* < 0.05), respectively (Table 1). Female whitefly adult longevity, oviposition days, and fecundity increased by 77.8%, 82.8%, and 75.1%, respectively, in the PX + BT treatment as compared with the BT treatment (*p* < 0.05). Similarly, these parameters increased by 36.1%, 43.4%, and 76.4%, respectively, in the PX − BT treatment as compared with the BT treatment (*p* < 0.05). The egg duration, male adult longevity, total preoviposition period (TPOP), and female proportion (Nf/N) ratio were not significantly different among the different treatments (*p* > 0.05) (Table 1).

The population parameters of *B. tabaci*, i.e., the finite rate of increase (*λ*), intrinsic rate of increase (*r*), net reproductive rate (*R*_0_), and mean generation time (*T*) are listed in Table 2. The *r*, *R*_0_, and *λ* increased by 26.3%, 131.3%, and 3.1% in the PX − BT treatment as compared to in the BT treatment (*p* < 0.05), respectively. The R_0_ notably increased by 88.6% in the PX + BT treatment as compared to in the BT treatment (*p* < 0.05). However, *T* did not show any significant difference among the different treatments (*p* > 0.05) (Table 2).

### 3.2. Life Table Analysis of B. tabaci

**Figure 2:**
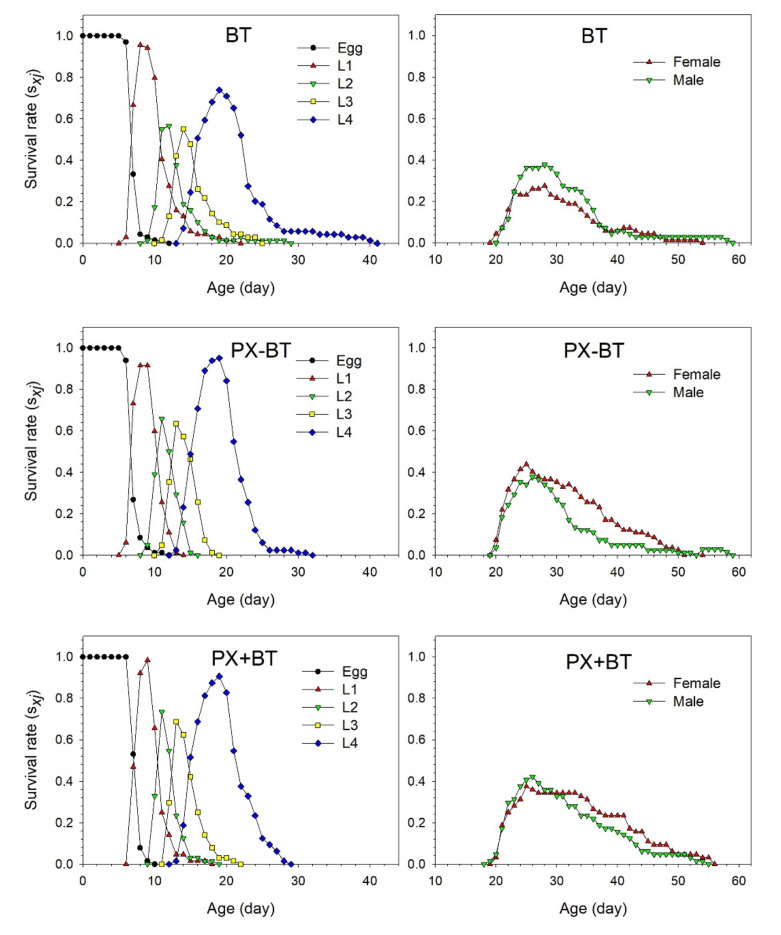
Age-stage specific survival rate (*s_xj_*) of *Bemisia tabaci* when feeding on Chinese kale alone (BT), with prior *Plutella xylostella* damage (PX − BT) or with simultaneous *P. xylostella* damage (PX + BT).

The age-stage specific survival rate (*s_xj_*) illustrated the probability that a *B. tabaci* egg can survive to age *x* and stage *j* in the three different treatments (Figure 2). Significant overlaps between stages were found among the three treatments of *B. tabaci* owing to variable developmental rates among the individuals. The survival rate of eggs or first nymphs reached more than 90% in the PX − BT and PX + BT treatments. The peak survival of the second and third nymphs was observed at 11 and 13 days, respectively, in the PX − BT and PX + BT treatments, respectively, one day earlier than that in the BT control group. The survival rate of the fourth nymphs had increased by 21.2% in the PX − BT treatment and by 16.7% in the PX + BT treatment as compared with the BT treatment. The peak survival of females in the PX − BT and PX + BT treatments was observed at 25 days, which was 3 days earlier than that in the control treatment. Similarly, the peak survival of males was observed at 26 days in the treatment groups, 2 days earlier than the control.

**Figure 3:**
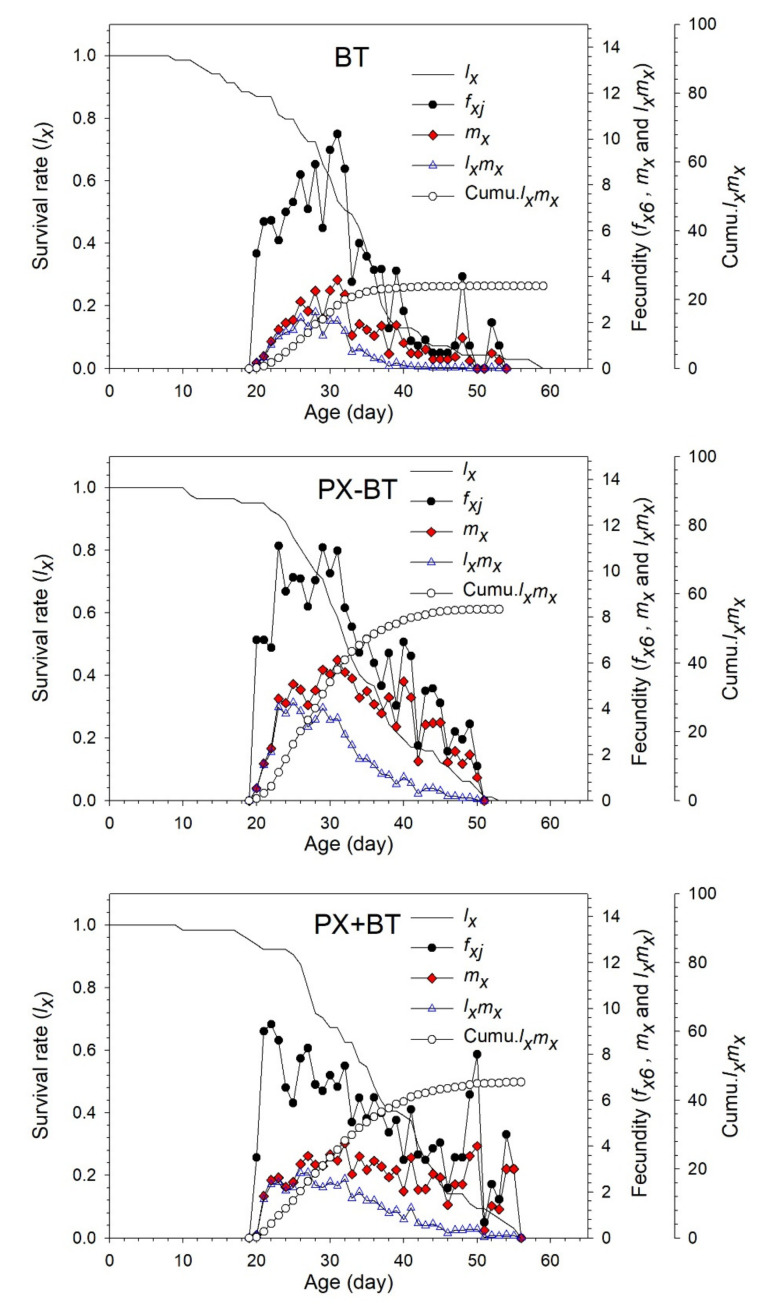
Age-specific survival rate (*l_x_*), female age-stage specific fecundities (*f_x_*), fecundity (*m_x_*) and net maternity (*l_x_m_x_*) of *Bemisia tabaci* when feeding on Chinese kale alone (BT), with prior *Plutella xylostella* damage (PX − BT) or with simultaneous *P. xylostella* damage (PX + BT).

The age-specific survival rate (*l_x_*), female age-stage specific fecundity (*f_x_*), age-specific fecundity (*m_x_*), age-specific net maternity value (*l_x_m_x_*) and cumulative *l_x_m_x_* value (Cumu.*l_x_m_x_*) of *B. tabaci* in the three different treatments were shown in Figure 3. The *l_x_* curve declined from 100% to 60.9% in the control BT treatment, from 100% to 63.4% in the PX − BT treatment, and from 100% to 67.2% in the PX + BT treatment in the first 30 days. After 30 days, the survival rate quickly dropped to zero. In the control BT treatment, the *f_x_* and *m_x_* curves reached reproductive peaks at 31 days of age, with the highest fecundity being 10 and 4 hatched eggs, respectively. However, in the PX − BT treatment, *f_x_* had three peaks, suggesting that the oviposition periods of *B. tabaci* were not concentrated due to DBM pre-infestation. The Cumu.*l_x_m_x_* values were 55.6 for the PX − BT treatment and 45.3 for the PX + BT treatment, which were 2.32 and 1.89 times those for the BT treatment, respectively.

**Figure 4:**
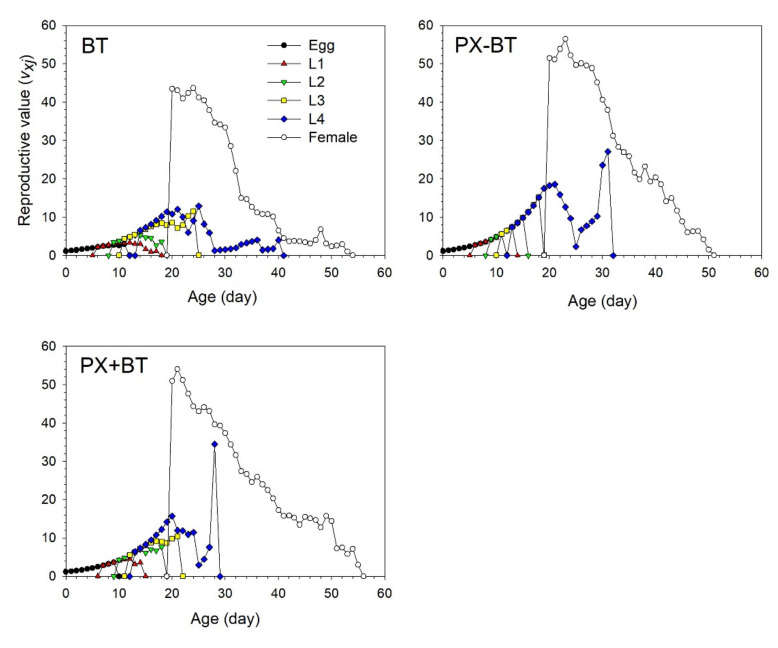
Age-stage specific reproductive value (*v_xj_*) of *Bemisia tabaci* when feeding on Chinese kale alone (BT), with prior *Plutella xylostella* damage (PX − BT) or with simultaneous *P. xylostella* damage (PX + BT).

The age-stage specific reproductive values (*v_xj_*) of *B. tabaci* represented the contribution of an individual at age *x* and stage *j* to the future population (Figure 4). The reproductive peaks of 43.5 and 43.7 occurred at 20 and 24 days of age, respectively, in the BT treatment. In contrast, the peaks of *v_xj_* that occurred at 23 and 21 days were as high as 56.5 and 53.9 eggs in the PX − BT and PX + BT treatments, respectively, which were significantly higher than the peaks in the BT treatment.

### 3.3. Performance of DBM

The first instar, third instar, pupa, and egg-adult (from embryonic development to adult emergence) developmental duration of DBM were 12%, 21.1%, 14.9% and 10.8% lower when feeding on plants that were simultaneously exposed to whiteflies plus DBM (BT + PX) (*p* < 0.05) (Table 3). In contrast, the fecundity of DBM was markedly increased by 31.7% with BT + PX treatment compared with the PX treatment (*p* < 0.05). However, the female adult longevity and oviposition days of DBM markedly decreased by 28.6% and 27.3%, respectively, after the BT − PX treatment (*p* < 0.05). Egg duration, male adult longevity, adult preoviposition period (APOP), TPOP, and sex ratio (Nf/N) were not significantly different among the different treatments (*p* > 0.05) (Table 3).

The net reproductive rate (*R*_0_) of DBM was greatly increased by 32.2% in the BT + PX treatment as compared with the PX treatment (*p* < 0.05). In contrast, the intrinsic rate of increase (*r*), finite rate of increase (*λ*), and mean generation time (*T*) of DBM were not significantly different among the BT − PX, BT + PX, and PX treatments (*p* > 0.05) (Table 4).

### 3.4. Life Table Analysis of DBM

**Figure 5:**
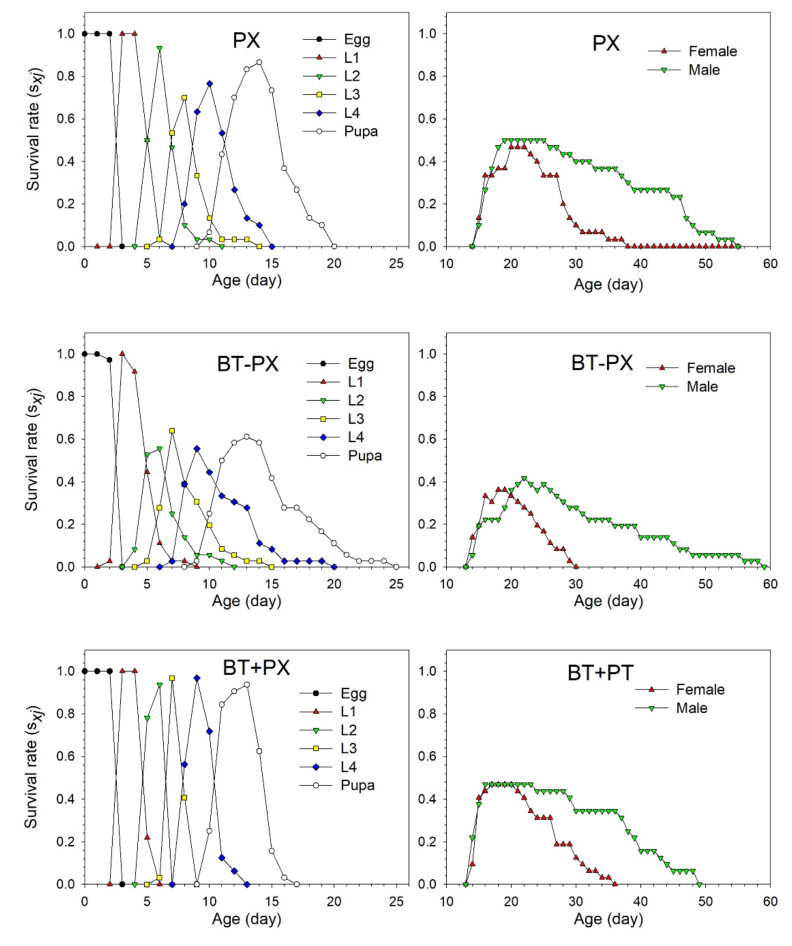
Age-stage specific survival rate (*s_xj_*) of *Plutella xylostella* when feeding on Chinese kale alone (PX), with prior *Bemisia tabaci* damage (BT − PX) or with simultaneous *B. tabaci* damage (BT + PX).

The age-stage specific survival rate (*s_xj_*) illustrated the probability that a DBM egg can survive to age *x* and stage *j* in the three different treatments (Figure 5). The survival rates of second, fourth instar larvae and pupae decreased by 37.8%, 21.1%, and 25.6%, respectively, in the BT − PX treatment as compared with the control PX treatment, and the pupal stage ended at 25 days, which was five days slower than that observed in the PX treatment. In contrast, the survival rates of third and fourth instar increased by 26.9% and 20.2%, respectively, in the BT + PX treatment as compared to the PX treatment, and the pupal stage ended at 17 days, which was three days faster than that in the PX treatment. The peak survival of females was observed at 18 and 17 days in the PX − BT (36.1%) and PX + BT treatments (46.9%) treatments, respectively, which were two to three days earlier than that in the PX treatment (46.7%). Similarly, the peak survival of males was observed at 16 days in the PX + BT treatment (46.9%), which was three days earlier than that in the PX treatment (50%). In contrast, the peak survival of males was observed at 22 days in the BT − PX treatment (41.7%), which was three days slower than that in the PX treatment.

**Figure 6:**
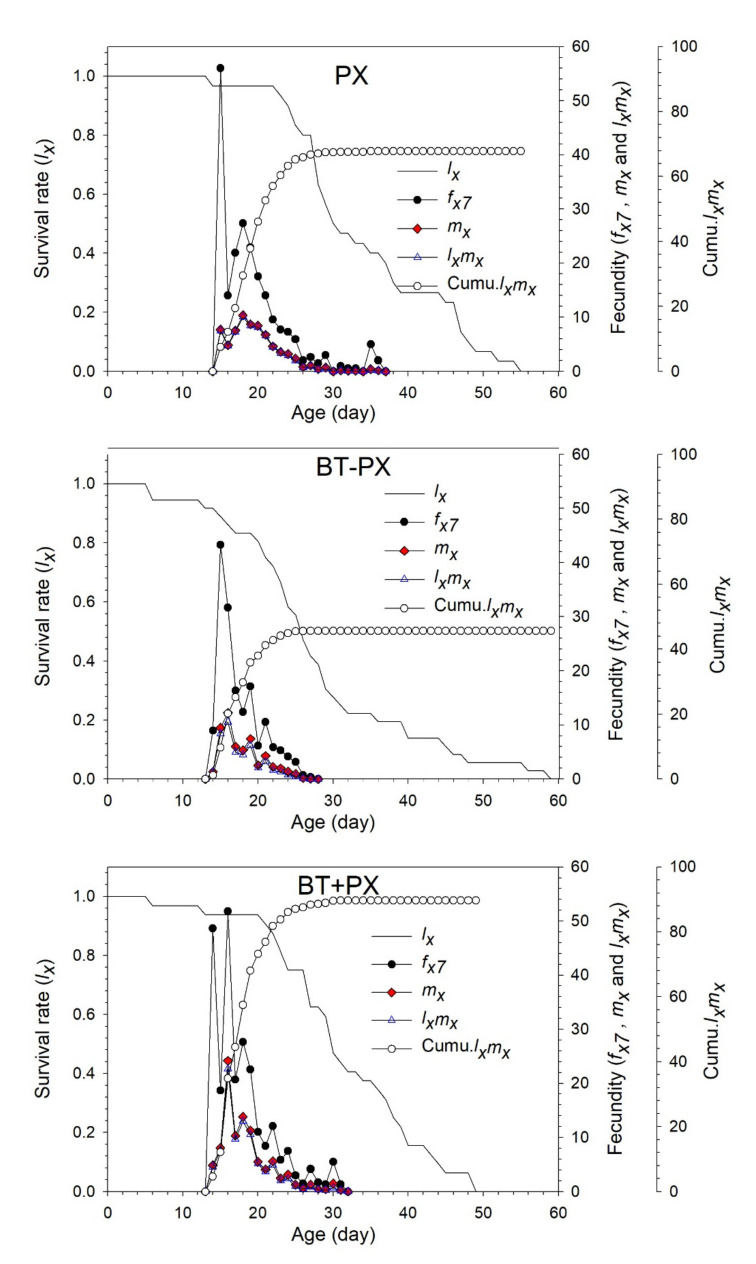
Age-specific survival rate (*l_x_*), female age-stage specific fecundities (*f_x_*), fecundity (*m_x_*) and net maternity (*l_x_m_x_*) of *Plutella xylostella* when feeding on Chinese kale alone (PX), with prior *Bemisia tabaci* damage (BT − PX) or with simultaneous *B. tabaci* damage (BT + PX).

The age-specific survival rate (*l_x_*), female age-stage specific fecundity (*f_x_*), age-specific fecundity (*m_x_*), age-specific net maternity value (*l_x_m_x_*), and cumulative *l_x_m_x_* value (Cumu.*l_x_m_x_*) of the DBM in the three different treatments were shown in Figure 6. The *l_x_* curve slowly declined from 100% to 90% in the first 24 days in the PX treatment, in the first 15 days in the BT − PX treatment, and in the first 21 days in the BT + PX treatment. Subsequently, the survival rate gradually decreased to zero. The *f_x_* curve reached a reproductive peak at 15 and 16 days of age, with the highest fecundity being 43 and 51 hatched eggs in the BT − PX and BT + PX treatments, respectively, which was lower than that in the PX treatment (at 15 days with 56). The *m_x_* reached a reproductive peak at 16 days of age, with the highest fecundity being 12 and 24 hatched eggs in the BT − PX and BT + PX treatments, respectively, which was higher than 10 in the PX treatment at 18 days. The Cumu.*l_x_m_x_* values were 45.6 in the BT − PX treatment and 89.7 in the BT + PX treatment, which were 0.67 and 1.32 times those of the PX treatment, respectively.

**Figure 7:**
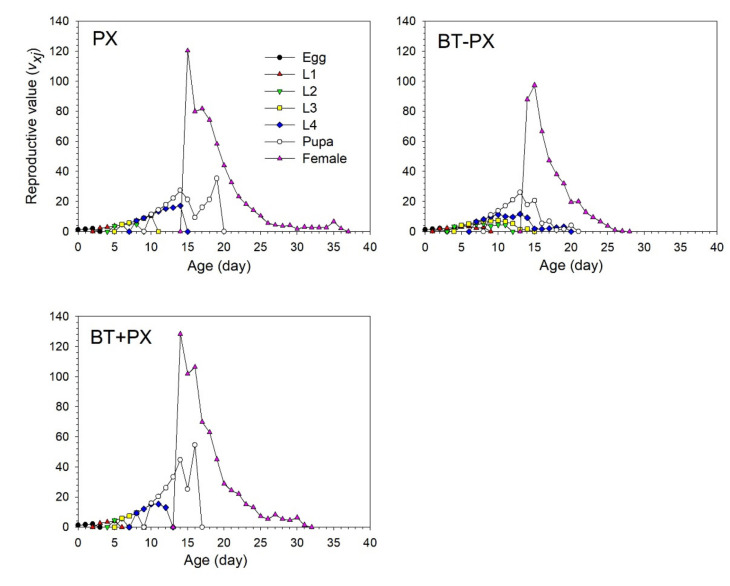
Age-stage specific reproductive value (*v_xj_*) of *Plutella xylostella* when feeding on Chinese kale alone (PX), with prior *Bemisia tabaci* damage (BT − PX) or with simultaneous *B. tabaci* damage (BT + PX).

The age-stage specific reproductive values (*v_xj_*) of DBM indicated the contribution of an individual at age *x* and stage *j* to the future population (Figure 7). The reproductive peaks occurred at 15 days of age, reaching a peak of 120.4 in the BT treatment. In contrast, the peaks of *v_xj_* that occurred at 15 and 14 days were 97.3 and 128 eggs in the BT − PX and BT + PX treatments, respectively.

## 4. Discussion

Different herbivores feeding on the same plant can interact through plant-mediated effects. Two important herbivore pests *B. tabaci* and DBM often co-occur in cruciferous vegetables. However, little is known about how they interact with each other. In the present study, we found support for our hypotheses that *B. tabaci* gain more fitness benefits when mixing with DBM on Chinese kale. Our results indicate that potential plant-mediated interactions occurred between *B. tabaci* and DBM feeding on Chinese kale. *Bemisia tabaci* gained fitness benefits in adult longevity, oviposition days, fecundity, and *R*_0_, *r*, and *λ* from pre-infestation with DBM, while female longevity and oviposition days of DBM were significantly reduced with pre-infestation with *B. tabaci*. Furthermore, co-infestation of *B. tabaci* and DBM was beneficial to the performance of the *B. tabaci* population. Therefore, when they mixed on the same host plant, the induced interactions favored *B. tabaci* as a superior competitor, which might be one of the reasons for the rapid expansion and outbreak of the whitefly population worldwide.

To date, some studies have investigated how infestation with prior herbivores affect the performance of subsequent herbivores. However, the results are highly variable. Phloem-feeding pest aphid pre-infestation has been found to have positive [13,15], neutral [31,32] or negative [33] impacts on leaf-chewing pest caterpillars that subsequently feed on the same plant. Recently, it has been reported that *Myzus persicae* (Suiz.) (Hemiptera: Aphididae) pre-infestation promotes the performance of the Colorado potato beetle *Leptinotarsa decemlineata* Say (Coleoptera: Chrysomelidae) [3]. In the present study, we found that pre-infestation with the cotton whitefly lowered the longevity and oviposition days of DBM, but DBM pre-infestation improved the performance and population growth of cotton whiteflies. Similar to our results, cotton whitefly pre-infestation also negatively affects the performance of *Trichoplusia ni* (Huebner) (Lepidoptera: Noctuidae) [34], *Liriomyza sativae* Blanchard (Diptera: Agromyzidae) [35], *M. persicae* [36], and *Pieris rapae* L. (Lepidoptera: Pieridae) [20]. However, the effects of heterospecific pre-infestation on cotton whitefly performance were inconsistent. The pre-infestation with *Heliothis* (Lepidoptera: Noctuidae) larvae favor the development of *B. tabaci* [37]. However, the pre-infestation with *Helicoverpa zea* (Boddie) (Lepidoptera: Noctuidae) larvae have no effect on *B. tabaci* survival and development [38]. Cotton spider mite *Tetranychus turkestani* Ugarov et Nikolski (Acari: Tetranychidae) pre-infestation reduces the population density of *B. tabaci* [39]. On the contrary, pre-infestation with the carmine spider mite *Tetranychus cinnabarinus* (Boisduval) (Acari: Tetranychidae) favors the performance of *B. tabaci*, but pre-infestation with the mealybug *Phenacoccus solenopsis* Tinsley (Hemiptera: Pseudococcidae) leads to the opposite effects [40]. These studies indicate that the plant defense response induced by *B. tabaci* first attacking healthy plants is unfavorable to the population growth of subsequent herbivores, which may help *B. tabaci* become a strong competitor. However, the effect of host plants first damaged by other herbivores on *B. tabaci* population may vary with insect species, and further research is needed.

In natural ecological systems, different insect species often simultaneously infest the same plant. Therefore, the co-occurrence of multiple herbivores may have different effects on the performance of herbivore pests. In the present study, the effects on the performance of *B. tabaci* and DBM were significantly different when they simultaneously infested Chinese kale; the egg-adult developmental duration of *B. tabaci* decreased, but the oviposition days, fecundity, and female longevity increased significantly compared with the case of *B. tabaci* infestation alone. In contrast, the pre-adult duration of the DBM decreased significantly, but the oviposition days, fecundity, and female longevity did not change significantly compared with the case of DBM infestation alone. These results suggest that co-infestation of DBM and *B. tabaci* might not only make the plant more susceptible to whiteflies but also facilitate the performance of the phloem feeder. It has been reported that *B. tabaci* feeding strongly represses DBM-induced transcriptional response when feeding together, which would lead to the repression of the production of defense chemicals indole glucosinolate [41]. This could be a reason why the whitefly developed better on plants damaged by caterpillars. Similar to our results, the aphid *Brevicoryne brassicae* (L.) (Hemiptera: Aphididae) performs well on black mustard plants co-infested with the butterfly *Pieris brassicae* L. (Lepidoptera: Pieridae) [13,42]. However, the butterfly *D. plexippus* benefits from aphid *A. nerii* feeding, while the aphid performance is impaired by butterfly feeding when co-occurring [15]. The damage by *L. decemlineata* negatively impacts *M. persicae* performance when they are feeding together [3]. In addition, plant-mediated interactions between aphid *B. brassicae* and DBM are density-dependent, i.e., the growth of DBM is increased at a low aphid density, whereas DBM feeding on plants colonized by aphids at a high density has a reduced growth rate [43]. Similar findings have been reported in *B. brassicae* and *P. brassicae* on *Brassica nigra* Koch plants [2]. The results obtained above indicate that sequences of arrival of different feeding guild herbivores, insect density, and host plant species might have an important impact on insect population dynamics and community structure.

The life table provides the most comprehensive information on the survivorship, development, and reproduction of a population [44,45]; therefore, it is widely used to evaluate the effects of biological (e.g., host plant species and plant nutrient level) and abiotic factors (e.g., temperature and chemical pesticides) on insect population growth and reproduction [46,47]. The population parameter *λ* summarizes the physiological qualities of an animal relative to its capacity to increase the population and is often used to evaluate the fitness of populations across diverse climatic and food-related conditions [48,49,50]. Therefore, the stronger the adaptability of an insect population, the higher the *r*-value. In the present study, DBM pre-infestation significantly increased the *r*-value of *B. tabaci*, and the *r*-value of *B. tabaci* also showed an increasing trend when they fed simultaneously with the DBM. In contrast, the *r*-value of DBM did not change significantly with *B. tabaci* pre-infestation or simultaneous infestation. To our knowledge, this is the first study to investigate plant-mediated effects on the performance and fitness of different pest insects using the life table method. More importantly, our results suggest that *B. tabaci* has become a dominant competitor when it is mixed with DBM on the same host plant irrespective of sequences of their arrival. Furthermore, the present study indicates that a combination of biological and ecological methods to study plant-mediated interactions between insect species may be conducive to a better understanding of the complex relationships in plant-insect associations.

## 5. Conclusions

*Bemisia tabaci* and DBM are some of the most destructive pests in the world, and they often occur together in cruciferous plants. We demonstrate that *B. tabaci* has become a dominant competitor by gaining increased fitness benefits when it is mixed with DBM on the same host plant irrespective of sequences of their arrival, which may be one of the reasons for the rapid expansion and outbreak of the whitefly population worldwide. These findings broaden our understanding of the population dynamics of herbivores belonging to different feeding guilds with various arriving sequences in nature. Further research is needed to explore interactions involving multiple herbivores and their natural enemies, which will greatly contribute to our understanding of plant responses and their consequences for trophic interactions and ecological communities in a broader ecosystem framework.

## Figures and Tables

**Table 1:** Developmental duration and reproduction (M ± SE) of *Bemisia tabaci* when feeding on Chinese kale alone (BT), with prior DBM damage (PX − BT) or with simultaneous DBM damage (PX + BT).

Stage	BT	PX − BT	PX + BT
Egg	7.4 ± 0.1 a	7.4 ± 0.1 a	7.6 ± 0.1 a
First instar	4.5 ± 0.2 a	3.6 ± 0.1 b	3.5 ± 0.1 b
Second instar	2.4 ± 0.1 a	2.1 ± 0.0 b	2.1 ± 0.1 ab
Third instar	2.9 ± 0.2 a	2.5 ± 0.1 b	2.6 ± 0.1 ab
Fourth instar	7.4 ± 0.3 a	6.7 ± 0.1 b	6.8 ± 0.1 ab
Egg-adult	24.1 ± 0.6 a	22.2 ± 0.2 b	22.6 ± 0.3 b
Female adult longevity	10.8 ± 1.2 c	14.7 ± 1.2 b	19.2 ± 1.6 a
Male adult longevity	11.8 ± 1.1 a	10.8 ± 1.2 a	12.8 ± 1.4 a
Oviposition days	9.9 ± 1.1 c	14.2 ± 1.2 b	18.1 ± 1.6 a
Fecundity (egg/female)	66.2 ± 9.6 b	116.8 ± 11.3 a	115.9 ± 10.9 a
TPOP	24.2 ± 1.0 a	22.3 ± 0.3 a	22.6 ± 0.4 a
Nf/N ratio	0.362 ± 0.06 a	0.476 ± 0.06 a	0.391 ± 0.06 a

TPOP represents total preoviposition period. Nf/N ratio represents female proportion. Means in the same row followed by different lowercases represent a significant difference among treatments (*p* < 0.05).

**Table 2:** Population parameters (M ± SE) of *Bemisia tabaci* when feeding on Chinese kale alone (BT), with prior DBM damage (PX − BT) or with simultaneous DBM damage (PX + BT).

Population Parameters	BT	PX − BT	PX + BT
Intrinsic rate of increase (*r*) (d^−1^)	0.11 ± 0.01 b	0.14 ± 0.01 a	0.13 ± 0.01 ab
Net reproductive rate (*R*_0_) (egg)	24.00 ± 5.15 b	55.54 ± 8.35 a	45.27 ± 8.20 a
Finite rate of increase (*λ*) (d^−1^)	1.12 ± 0.01 b	1.15 ± 0.01 a	1.14 ± 0.01 ab
Mean generation time (*T*) (d)	28.59 ± 0.42 a	28.59 ± 0.39 a	29.62 ± 0.54 a

Means in the same row followed by different lowercases represent a significant difference among treatments (*p* < 0.05).

**Table 3:** Developmental durations and reproduction (M ± SE) of *Plutella xylostella* when feeding on Chinese kale alone (PX), with prior whitefly damage (BT − PX) or with simultaneous whitefly damage (BT + PX).

Stage	PX	BT − PX	BT + PX
Egg	3.0 ± 0.0 a	3.0 ± 0.0 a	3.0 ± 0.0 a
First instar	2.5 ± 0.1 a	2.5 ± 0.2 ab	2.2 ± 0.1 b
Second instar	2.1 ± 0.1 a	1.8 ± 0.1 a	1.8 ± 0.1 a
Third instar	1.8 ± 0.1 b	2.1 ± 0.1 a	1.5 ± 0.1 c
Fourth instar	2.7 ± 0.1 a	2.8 ± 0.1 a	2.5 ± 0.1 a
Pupa	4.7 ± 0.1 a	4.7 ± 0.1 a	4.0 ± 0.1 b
Egg-adult	16.7 ± 0.3 a	16.8 ± 0.5 a	14.9 ± 0.1 b
Female adult longevity	11.9 ± 1.0 a	8.5 ± 0.7 b	12.6 ± 1.2 a
Male adult longevity	24.8 ± 2.1 a	19.1 ± 2.8 a	23.6 ± 1.7 a
Oviposition days	8.8 ± 0.8 a	6.4 ± 0.5 b	7.7 ± 1.1 ab
Fecundity(egg/female)	145.4 ± 16.9 ab	109.4 ± 20.9 b	191.3 ± 29.9 a
APOP	0.6 ± 0.2 a	0.4 ± 0.2 a	1.5 ± 0.6 a
TPOP	17.4 ± 0.6 a	16.3 ± 0.5 a	16.3 ± 0.6 a
Nf/N ratio	0.5 ± 0.1 a	0.4 ± 0.1 a	0.5 ± 0.1 a

APOP represents adult preoviposition period; TPOP represents total preoviposition period; Nf/N ratio represents female proportion. Means in the same row followed by different letters represent a significant difference among treatments (*p* < 0.05).

**Table 4:** Population parameters (M ± SE) of *Plutella xylostella* when feeding on Chinese kale alone (PX), with prior whitefly damage (BT − PX) or with simultaneous whitefly damage (BT + PX).

Population Parameters	PX	BT − PX	BT + PX
Intrinsic rate of increase (*r*) (d^−1^)	0.22 ± 0.01 a	0.21 ± 0.01 a	0.25 ± 0.02 a
Net reproductive rate (*R*_0_) (egg)	67.83 ± 9.29 b	45.58 ± 8.35 b	89.69 ± 11.74 a
Finite rate of increase (*λ*) (d^−1^)	1.24 ± 0.01 a	1.23 ± 0.02 a	1.28 ± 0.02 a
Mean generation time (*T*) (d)	19.52 ± 0.58 a	18.10 ± 0.61 a	18.28 ± 0.50 a

Means in the same row followed by different letters represent a significant difference among treatments (*p* < 0.05).

## Data Availability

All data generated or analyzed during this study are included in this published article.
